# Ubiquity of Kelvin–Helmholtz waves at Earth's magnetopause

**DOI:** 10.1038/ncomms8019

**Published:** 2015-05-11

**Authors:** Shiva Kavosi, Joachim Raeder

**Affiliations:** 1 Department of Physics and Space Science Center, University of New Hampshire, 8 College Road, Durham, New Hampshire 03824, USA

## Abstract

Magnetic reconnection is believed to be the dominant process by which solar wind plasma enters the magnetosphere. However, for periods of northward interplanetary magnetic field (IMF) reconnection is less likely at the dayside magnetopause, and Kelvin–Helmholtz waves (KHWs) may be important agents for plasma entry and for the excitation of ultra-low-frequency (ULF) waves. The relative importance of KHWs is controversial because no statistical data on their occurrence frequency exist. Here we survey 7 years of *in situ* data from the NASA THEMIS (Time History of Events and Macro scale Interactions during Substorms) mission and find that KHWs occur at the magnetopause ∼19% of the time. The rate increases with solar wind speed, Alfven Mach number and number density, but is mostly independent of IMF magnitude. KHWs may thus be more important for plasma transport across the magnetopause than previously thought, and frequently drive magnetospheric ULF waves.

## Abstract (web summary)

Although magnetic reconnection is recognized as the dominant mode for solar wind plasma to enter the magnetosphere, Kelvin–Helmholtz waves (KHW) have been suggested to also be involved. Here, the authors use 7 years of THEMIS data to show that KHW occur 19% of the time, and may be important for plasma transport.

## Graphical abstract

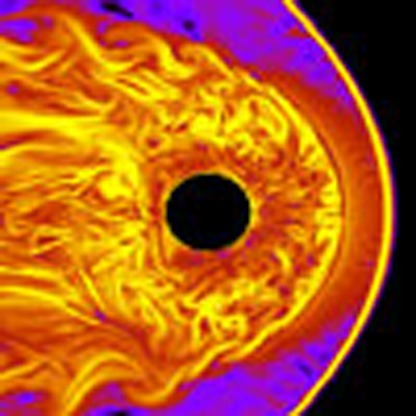

The magnetosphere is filled with plasma from the ionosphere and the solar wind (SW). Although ionosphere plasma can be easily traced by its composition, the entry pathways of SW plasma are much less clear. Early models suggested magnetic reconnection at the dayside magnetopause (MP) and subsequent convection into the tail as the main path^1^,^2^. However, later research showed that the SW plasma density in the tail often maximizes during times of northward interplanetary magnetic field (IMF), forming the Cold Dense Plasma Sheet. As magnetic reconnection does not occur during northward IMF at the dayside MP, and because of the Cold Dense Plasma Sheet plasma properties, other entry mechanisms must be involved in the entry process. High-latitude magnetic reconnection near the cusps^3^,^4^, impulsive penetration^5^, gradient drift^6^, particle diffusion^7^ and the Kelvin–Helmholtz instability (KHI)^8^,^9^,^10^ have been suggested as viable mechanisms operating during northward IMF. Although cusp reconnection and Kelvin–Helmholtz waves (KHWs) have received much attention recently^3^,^11^, the relative importance of all these processes remains unknown^7^. Although KHWs may play an important role as a SW plasma entry mechanism, they are also considered drivers of magnetosphere ultra-low-frequency (ULF) waves^12^,^13^, which in turn strongly affect the radiation belts^14^.

KHWs have been studied extensively using *in-situ* data and simulations. Event studies have shown that KHWs occur at times at the MP, and have revealed some of their basic properties^10^,^11^,^15^,^16^. Both magneto-hydrodynamic (MHD) and kinetic simulations, mostly in a simplified two-dimensional geometry, have shown how KHWs can roll up and mix SW plasma with magnetosphere plasma^17^,^18^. More recently, global MHD simulations of the magnetosphere have shown the development of KHWs^19^. Despite the progress in understanding KHWs properties and their effect on transport, little is known about their occurrence rate. Linear theory^20^ suggests that KHWs are most unstable at high-flow shear, for example, high solar wind speed, and when the IMF is nearly northward, for example, nearly parallel to the magnetosphere field. As these conditions occur rarely together, KHWs have often been considered infrequent events.

Because satellite orbital dynamics makes it impossible to monitor the MP over long-time periods, in the past only intermittent observations of the MP were available. However, the THEMIS (Time History of Events and Macro scale Interactions during Substorms) mission, originally designed to study substorms^21^, has almost ideal equatorial orbits to study KHWs. With orbit apogees between 12 RE (Earth radii) and 30 RE, the spacecraft frequently cross the MP flanks during the spring and fall seasons, as the orbits undergo precession around the Earth.

We survey the THEMIS data to obtain a database of MP crossings, and identify crossings where KHWs are present. The statistical analysis shows that KHWs occur ∼19% of the time regardless of the solar wind conditions. We find that the KHWs occurrence rate increases with solar wind speed, Alfven Mach number and number density, but is mostly independent of IMF magnitude. The occurrence rate increases with IMF cone angle and maximizes at zero IMF clock angle. We find that KHWs also occur at higher rate than expected for southward IMF. We conclude that KHWs may thus be more important for plasma transport across the MP than previously thought, and frequently drive magnetospheric ULF waves.

## Results

### Occurrence rate and IMF dependence

The duration of MP encounters can last from minutes to hours. To obtain occurrence rates, we divided each encounter into 5-min intervals. Each interval is classified as KHW or not, and tagged with ancillary data, such as time-shifted SW and IMF data. Our database (Supplementary Data 1) consists of ∼11,500 5-min samples, covering ∼960 h dwell time at the MP. The samples are nearly evenly divided between northward (∼500 h) and southward (∼460 h) IMF conditions.

**Figure 1:**
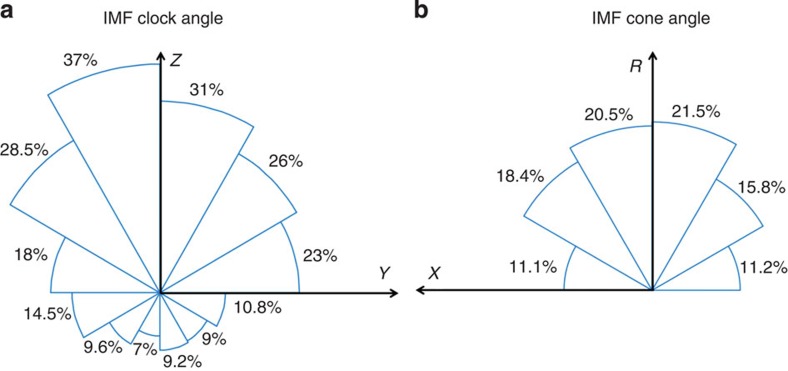
KHW occurrence rate as a function of IMF clock angle and cone angle. The clock angle is defined as atan(B_y_/B_z_), and the cone angle is defined as acos(*B*_X_/*B*). *X* points towards the Sun, *Y* points duskward, *Z* points north, and *R*=(*Y*^2^+*Z*^2^)^1/2^. KHW occurrence maximizes for northward IMF, but is still significant during southward IMF (**a**). The IMF is more effective generating KHW when it is oriented perpendicular to the Sun–Earth line (**b**).

We find that about half of the crossings show waves or quasi-periodic variations, but not all of them are KHWs. Figure 1 shows the KHWs occurrence rate as a function of IMF clock angle and IMF cone angle. As a function of clock angle, the occurrence rate is ∼35% for near northward IMF, near 20% if the IMF lies in the equatorial plane, and about 10% for southward IMF. The fact that KHWs occur during southward IMF at a significant rate is not expected; because it is generally thought that magnetic reconnection dominates over KHI during such conditions and prevents KHWs growth. The IMF cone angle dependence is as expected from the linear dispersion relation of KHWs^20^, which predicts that the instability maximizes when the magnetic field on either side of the shear layer is close to collinear, which occurs for ∼90° cone angle. The overall occurrence rate of KHWs is ∼19% regardless of solar wind and IMF conditions. This is a substantially higher rate than the linear dispersion relation would suggest.

### Solar wind parameter dependence

**Figure 2:**
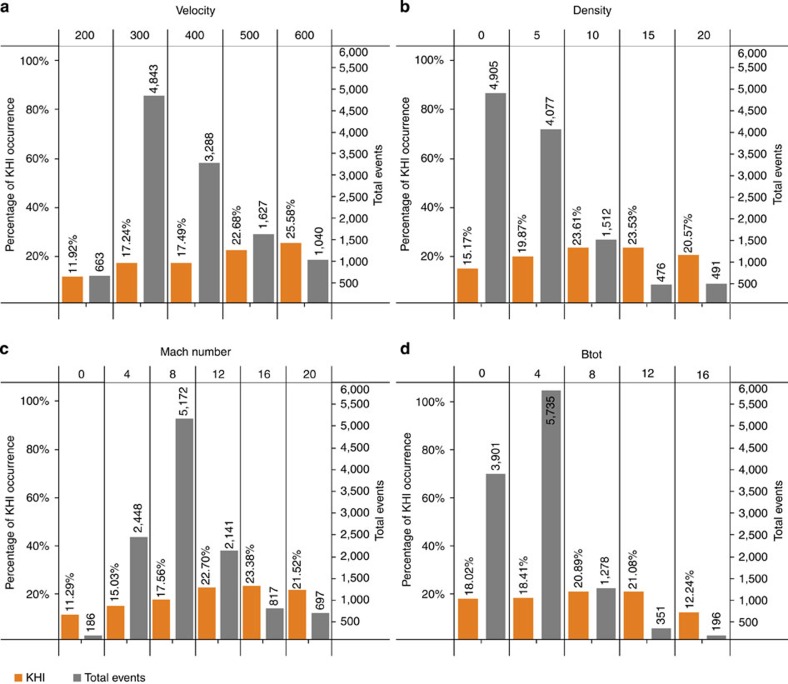
Occurrence rate of KHW as a function of solar wind plasma parameters. Orange bins show the relative KHW occurrence rate and grey bins show the number of 5-min KHW intervals in that bin. The panels show, respectively, the dependence on the solar wind velocity (**a**), the solar wind density (**b**), the solar wind Mach number (**c**) and the IMF magnitude. The parameter dependence is mostly as expected from the Kelvin-Helmholtz dispersion relation, but the significant occurrence rate at low velocity (<300 km s^−1^) is not expected.

Figure 2a shows occurrence percentage of KHWs (orange bins) and the corresponding number of 5-min intervals (grey bins) as a function of solar wind speed. The latter is shown to assess the statistical significance of the data. As expected, the occurrence frequency increases with solar wind speed. However, the occurrence of KHWs at very low solar wind speed is unexpected. There appears to be no low-speed cutoff for KHW; KHW are still observed at 270 km s^−1^ solar wind speed. The KHWs dependence on solar wind density (Fig. 2b) is weak. At low densities, there is a positive correlation, which tapers out for densities that are larger than 10 cm^−3^. There is also a positive correlation with the solar wind Alfven Mach number (Fig. 2c), which also tapers out at high (>16) Mach numbers. The IMF magnitude (Fig. 2d) appears only to have an effect for unusual high values of more than 16 nT. It is tempting to compare the KHWs dependencies with the dispersion relation for KHWs; however, the solar wind parameters are not the same as the plasma and field parameters on the magnetosheath side of the MP flanks. In particular, the solar wind is slowed down by the bow shock and then re-accelerates along the flanks of the magnetosphere. Therefore, the magnetosheath velocity is generally slower than the solar wind, but it is also possible that the draped IMF accelerates the magnetosheath plasma to speeds larger than the solar wind speed. However, the trends shown in Fig. 2 are in agreement with linear theory, in particular the increase of the KHW rate with solar wind speed, and the apparent suppression of KHWs for strong IMF, which was predicted by theory^22^.

## Discussion

Linear MHD theory predicts that KHWs are most unstable when the magnetic field on either side of the shear layer is perpendicular to both the flow direction and the direction of the velocity gradient. Furthermore, the growth rate increases with flow shear. Thus, it has commonly been assumed that KHWs at Earth's MP are restricted to times of nearly northward IMF and high solar wind speed. This would make them rare events with little importance for magnetospheric dynamics. Although the dispersion relation does not distinguish between northward and southward IMF, that is, whether the magnetic field is parallel or anti-parallel across the shear layer, it was commonly assumed that during southward IMF periods magnetic reconnection would dominate over KHW generation. However, recent reports have shown that KHWs may also occur during southward IMF conditions^23^,^24^, but these were case studies that give no indication as to whether these were singular events or whether they would occur more common. We find that the southward KHW events in our database are generally shorter (20 min on average) than those observed during northward IMF (45 min on average). The relative short duration of KHW events during southward IMF may explain why only few such events have been reported.

Refined theoretical analysis that has taken into account the finite width of the shear layer and its structure has further narrowed the parameter range under which KHWs should occur^25^. However, the true occurrence rate of KHWs remained uncertain, and many researchers assumed they were rare events. Statistical studies have long been hampered by the lack of suitable data. Although Pc5 waves observed on the ground are often associated with KHWs, they may also have other causes and thus provide no suitable statistics. On the other hand, *in situ* observations are restricted by satellite orbital dynamics. Before THEMIS, most missions had orbits that would preclude frequent KHW observations, or the missions were too short to obtain sufficient data for statistical studies. THEMIS has for the first time provided a sufficiently large database of MP crossings in the equatorial plane, together with suitable instrumentation, to allow for the study presented here.

Our results show that KHWs are much more ubiquitous and occur under most SW and IMF conditions. We confirm the presence of KHWs even during southward IMF conditions, in line with recent event studies^23^,^24^. During northward IMF, KHWs occur frequently, and particularly also during periods of very low solar wind speed. Theoretical models suggest that the growth rate diminishes for small flow shear and that there even may be a cutoff velocity. By contrast, we find only weak velocity dependence and no indication of a cutoff.

**Figure 3:**
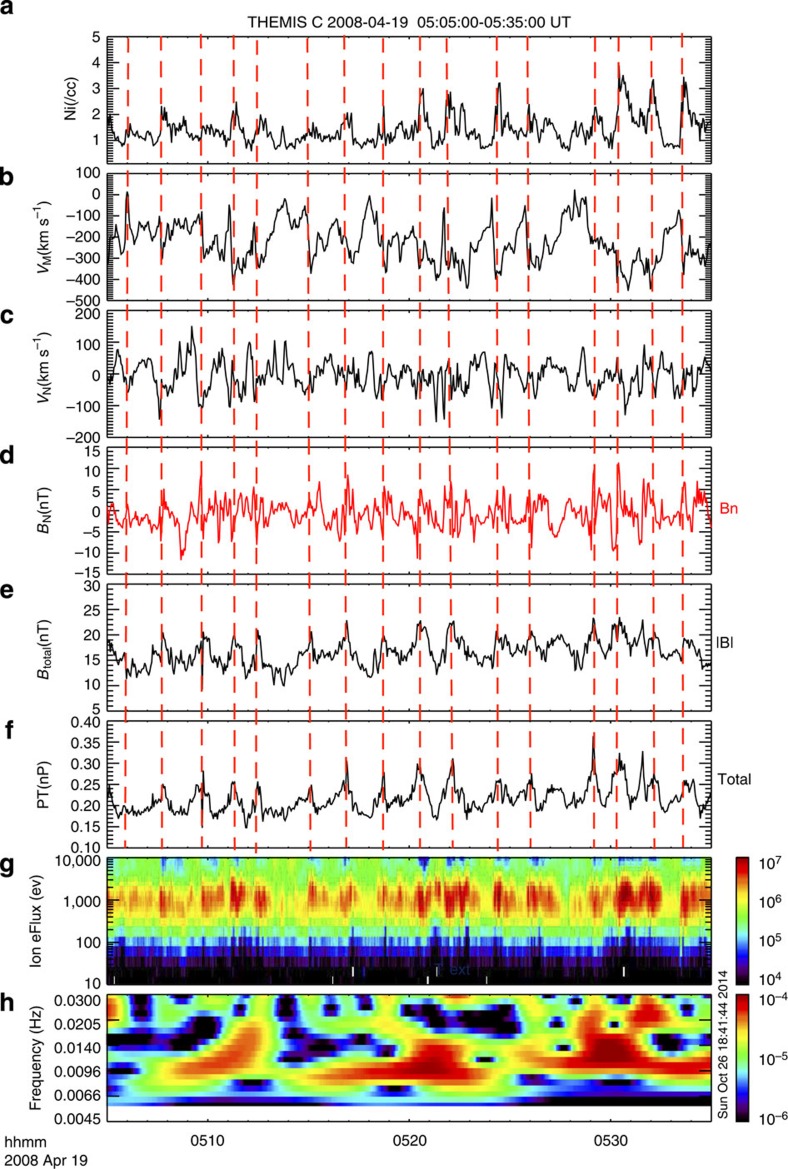
Kelvin–Helmholtz waves observed at the dawn flank magnetopause by THEMIS C on 19 April 2008. The panels show, from top to bottom: (**a**) the ion number density, (**b**) the M component of ion velocity, (**c**) normal component of the ion velocity vector, (**d**) normal component of magnetic field, (**e**) total magnetic field, (**f**) total (magnetic plus ion) pressure, (**g**) omnidirectional ion energy spectrogram and (**h**) wavelet spectra of the total pressure. The wave period is approximately 100 s.

It is not clear why the data are difficult to reconcile with linear theory, but the most likely reason seems to be that the dispersion relations are based on the assumption of a simply structured shear layer, that is, either a jump-like discontinuity or a smooth transition of finite thickness. In reality, however, the MP often has a complicated boundary layer structure, which generally does not match these assumptions. The presence of such boundary layers makes it thus difficult to test the dispersion relations, because single spacecraft observations generally do not reveal their structure. This is evident, for example, in the event shown in Fig. 3. During this KHW event, neither the magnetosheath plasma velocity nor the plasma density is well defined, and both vary over a large range of values.

Because KHWs can facilitate the entry of SW plasma into the magnetosphere^9^,^10^, they may be more important for the magnetosphere mass budget than previously thought. It has been shown that Kelvin–Helmholtz vortices can efficiently mix plasma. The breaking vortex greatly increases the area through which plasma can diffuse, and it also creates sharp gradients that make it possible for particles to cross from the magnetosheath to the magnetosphere because of finite Larmor radius effects. There is also evidence that magnetic reconnection may occur on small scales within the vortices^9^, which would also enable plasma transport across the boundaries. However, quantifying the importance of KHWs for mass transport across the MP remains to be done.

KHWs are also thought to be significant drivers of magnetospheric ULF waves, which in turn can energize the particles in Earth's radiation belts^12^,^13^,^14^. However, such ULF waves can also be generated through other different processes, such as solar wind buffeting or drift-resonant instabilities^26^. Thus, a reassessment of the importance of KHWs for radiation belt dynamics may be necessary.

## Methods

### Event selection

We surveyed data from 2007 to 2013, when the THEMIS spacecraft frequently crossed the MP during the dawn and dusk orbital phases^27^. We examined the plasma and magnetic field data to catalogue MP crossings with the motivation to identify KHWs. The magnetic field measurements were provided by the FGM instrument^28^ and plasma measurements were from the ESA spectrometer^29^. Figure 3 shows a typical example of a crossing where KHWs were present. We show the THEMIS magnetic field and velocity components in boundary normal coordinates (L,M,N)^30^ to facilitate the characterization of the oscillations. Different regions and waves were most easily identified in the ion energy spectra, shown in Fig. 3g). Oscillations occurred, where the probe C observed alternately hot magnetosphere plasma and cold magnetosheath plasma. The oscillations were also visible in the magnetic field normal component, *B*_N_ (Fig. 3d), the M and N components of the smoothed velocity (Fig. 3b and c), the ion number density (Fig. 3a) and the total pressure (magnetic plus ion pressure; Fig. 3f). The oscillations of the velocity normal component, *V*_N_, show that the MP moved back and forth. As the spacecraft orbital velocity was slow compared with *V*_N_, the oscillations must have been caused by MP surface waves. However, not all MP surface waves are the result of KHI. Other mechanisms can also lead to the excitation of surface waves, such as dynamic pressure variations in the solar wind or magnetosheath^31^, non-steady MP reconnection that can generate bulges in the MP or Flux Transfer Events (FTEs)^30^. We thus needed to discriminate all MP wave observations against FTEs and buffeting of the magnetosphere by the solar wind. We inspected solar wind data for every event, where possible, to confirm that the event was not preceded by rapid or periodic SW pressure changes^31^,^32^,^33^ that may have caused buffeting. Such events only produce a single bipolar *B*_N_ and are thus easily distinguishable from surface waves and ruled out by our requirement of at least four wave periods. They can also often be ruled out by their irregular structure, because KHWs are to large degree monochromatic wave trains. In the initial linear stage of the KHWs, the MP can be approximated by a planar surface, and thus there are no significant total pressure variations across the MP^11^,^34^,^35^. An example of such linear KHWs (Supplementary Fig. 1) shows that KHWs in the linear stage can be easily distinguished from FTEs by the absence of bipolar *B*_N_ signatures, and by the absence of maxima in |B| and the total pressure.

### Discrimination between KHW and FTEs

As KHWs in the nonlinear stage have some similar characteristics as FTEs, such as bipolar *B*_N_ and possibly similar wave periods of a few minutes, they may be difficult to differentiate. The properties of FTEs are well known^36^,^37^. FTEs are magnetic flux ropes whose magnetic signatures include a distinctive bipolar excursion in the magnetic field component *B*_N_ normal to the MP surface, either enhancements or crater-like variations of the magnetic field strength at the event centre^38^,^39^,^40^, and a deflection of the tangential *B*_L_ and *B*_M_ components, as shown in Supplementary Fig. 2. The bipolar FTE signature is brief (0.5–2 min) and sequences of FTEs are separated by longer periods of quiet, typically 3–8 min (refs 36, 37, 38, 39, 40, 41), which is summarized in Supplementary Table 1, whereas KHWs are continuous wave trains. In addition, the total (thermal and magnetic) pressure in a FTE typically maximizes at the centre of the event^37^ as can be seen in Supplementary Fig. 3. By contrast, in a KHW in the nonlinear stage, that is, within a rolled-up vortex the total pressure is expected to have a minimum at the centre and a maximum at the edge of the vortex^11^,^34^,^35^,^42^,^43^.The pressure minimum occurs because the centrifugal force of the rotating vortex pushes the plasma radially outward, as depicted in Supplementary Fig. 4. This figure also shows that there is a density jump at the edges of the vortices where the pressure should maximize. Figure 3 and Supplementary Fig. 5 show examples of nonlinear KHWs where the density jumps from magnetospheric to magnetosheath values indeed closely coincide with total pressure maxima at the edges of the vortices.

**Figure 4:**
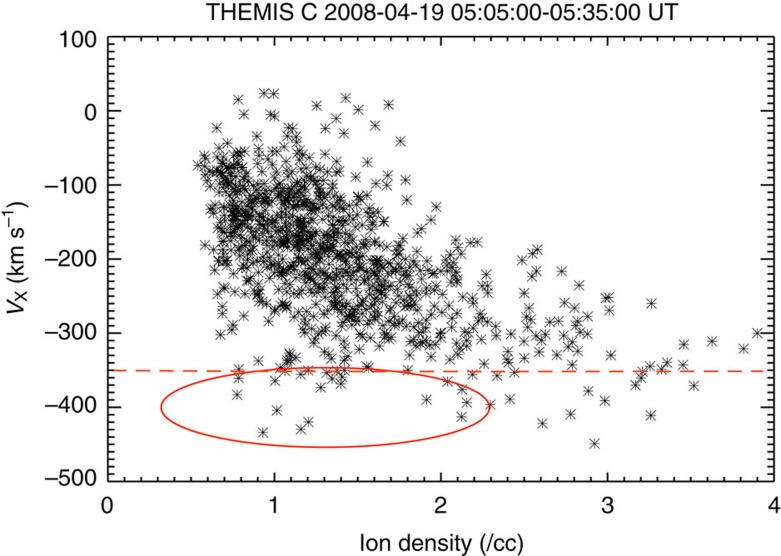
Scatter plot of the ion velocity *V*_X_ component versus ion density. The data are from THEMIS C for the 19 April 2008 event. Each symbol represents one of the samples, which were taken at 5 min cadence. Negative *V*_X_ values indicate anti-sunward flow. The plot confirms that this event consists of rolled-up Kelvin-Helmholtz vortices, because a fraction of the low-density magnetospheric plasma, indicated by the red ellipse, flows faster than the magnetosheath plasma (<−350 km s^−1^, shown by the dashed red line).

The above criteria, which are summarized in Supplementary Table 2 were not always sufficient to differentiate FTEs from KHWs. Therefore, we also exploited the fact that a rotating KHW vortex accelerates the plasma. When the KHW enters the nonlinear phase, at some distance *r*_c_ from the vortex centre, the centrifugal force *ρVϕ*^2^/*r*_c_ should be nearly equal for both denser and less dense media, where *ρ* is the plasma mass density and *Vϕ* is the azimuthal flow velocity; otherwise the vortex would disintegrate^44^,^45^. Thus, the less dense part of the vortex rotates faster than the denser part. Such low density, accelerated magnetosphere plasma can be exposed in a *ρ* (or number density *N*) versus *V*_X_ scatter plot, where the KHW or vortex, exhibits a distinct pattern^45^,^46^,^47^. This is demonstrated in Supplementary Fig. 6, where simulations have been used to create the expected scatter plot patterns. Figure 4 shows such a V_X_–N scatter plot generated from THEMIS C observations of the KHW example presented in Fig. 3. Concurrent Themis B observations in the magnetosheath showed plasma with ∼350 km s^−1^ velocity and ∼8 cm^−3^ density. Figure 4 shows that for part of the low-density (<4 cm^−3^) boundary layer ions, |V_X_| is larger than that of the high-density magnetosheath ions (*V*_X_∼−350 km s^−1^), which is due to the vortex rotation and is not expected for a FTE. Supplementary Fig. 7 shows that no such signature occurs for linear KHWs, that is, the case shown in Supplementary Fig. 1. Supplementary Fig. 8 shows the V_X_–N scatter plot for the FTEs presented in Supplementary Fig. 3. The pattern is clearly different from that produced by KHWs in the nonlinear stage, such as the one shown in Fig. 4, and can be used to distinguish them. However, this method could only be used for cases with northward IMF, because during southward IMF low-density, high-speed flows can also result from reconnection^11^. Supplementary Fig. 9 shows a case of KHWs during southward IMF. In this case, we ruled out FTEs because there are no distinct pressure maxima, and neither are there maxima or crater-like structures in the magnetic field magnitude. Instead, the magnetic field magnitude shows distinct minima, which would not be present at FTEs.

Supplementary Fig. 10 shows a unique case where FTEs and KHWs occur back-to-back. The corresponding scatter plot (Supplementary Fig. 11) shows that during the FTE interval, the tangential flows are mostly less than 100 km s^−1^, as opposed to the faster tangential flows during the KHW interval. However, the normal flow component (*V*_N_ in Supplementary Fig. 10) is much larger for the FTEs than for the KHWs. THEMIS A and THEMIS D also observed this event (not shown here). THEMIS A was located at (3.2,−10.0,3.4) at the beginning of the interval, that is, very close to THEMIS E. It observed essentially the same signatures as THEMIS E. THEMIS D, on the other hand, was located at (3.9,−9.8,3.7), that is, further on the magnetosheath side, and only observed the FTEs, but not the KHWs. This implies that the fluctuations in each sub-interval are of different nature. In particular, the amplitude of the KHW must be smaller than the size of the FTEs.

Whenever an event still remained ambiguous, we considered it not to be a KHW.

## Author contributions

J.R. initiated the study and guided the graduate student S.K., who screened the data and obtained the statistics. Both authors discussed the methods, results and implications at all stages.

## Additional information

**How to cite this article:** Kavosi, S. and Raeder, J. Ubiquity of Kelvin–Helmholtz waves at Earth's magnetopause. *Nat. Commun.* 6:7019 doi: 10.1038/ncomms8019 (2015).

## Supplementary Material

Supplementary Information Supplementary Figures 1-11, Supplementary Tables 1-2 and Supplementary References

Supplementary Data 1 Database of THEMIS magnetopause events used in this study. The columns provide the date, start and end time, probes that observed the event, and the classification of the event, where KH indicates that Kelvin-Helmholtz waves were observed, and MPC indicates a magnetopause crossing without KH waves.
