# Supplementary material for: Ubiquity of Kelvin–Helmholtz waves at Earth's magnetopause
Source: Nat Commun. 2015 May 11;6:7019. doi: 10.1038/ncomms8019 (PMC4432594; doi:10.1038/ncomms8019)
Supplement: Supplementary Information — Supplementary Figures 1-11, Supplementary Tables 1-2 and Supplementary References [file ncomms8019-s1.pdf]

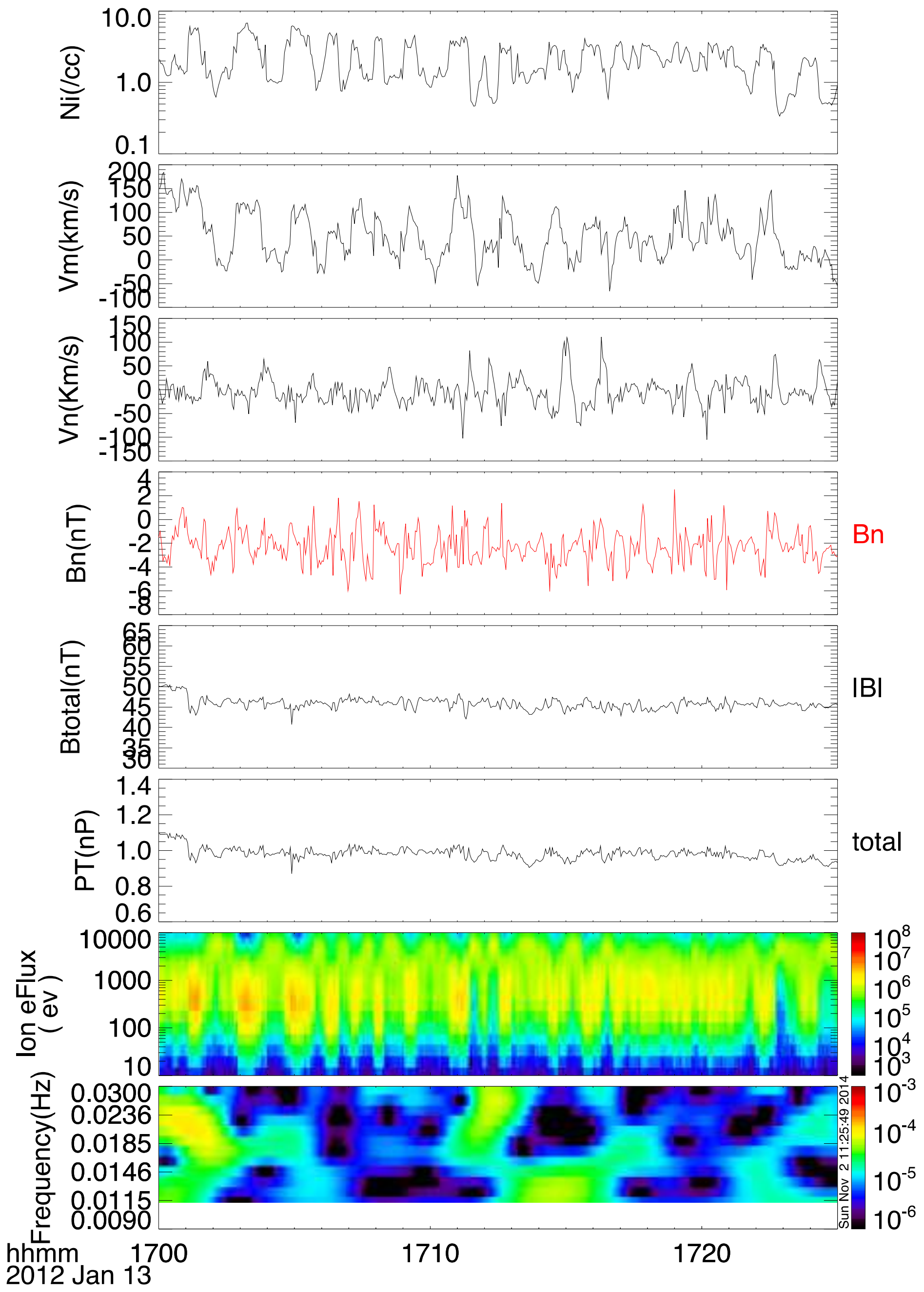

Supplementary Figure 1. THEMIS E observations of KHWs in linear stage on 13 Jan 2012. From top to bottom: (a) Ion density, (b) M component of the velocity V_M, (c) N components of the velocity V_N, (d) N component of magnetic field B_N, (e) Magnetic field magnitude |B|, (f) total (magnetic plus ion) pressure, (g) ion energy flux spectrogram, and (h) wavelet spectrum of the total pressure. The solar wind had a flow speed 450 km/s and density N = 13 cm^{-3}. The IMF vector was (1,2,4) nT. There were no significant solar wind dynamic pressure variations before or during the event. Themis E was located at (8.3,-7.6,3.4) and was moving sunward. Themis E observed quasi-periodic fluctuations at the dawn flank magnetopause during the interval 1640 - 1720 UT, but no significant fluctuations in total pressure or magnetic field magnitude. We thus conclude that this wave train is a KHW in the linear stage and it has not developed to a vortex yet.

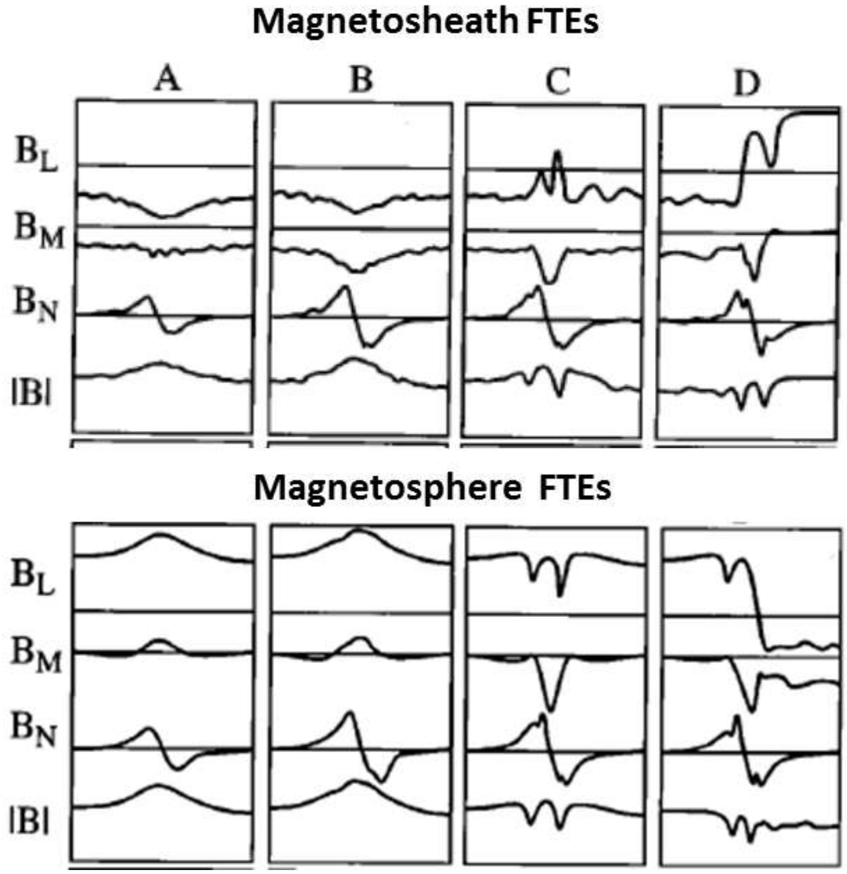

Supplementary Figure 2. FTE taxonomy. The panels illustrate the magnetic field signatures in boundary normal coordinates (B_L, B_M, B_N and IBI) for magnetosheath and magnetosphere FTEs. There are four main taxa: A, B, C and D, which differ by the distance of the observing spacecraft from the magnetopause, with A being farthest from the magnetopause, and D being closest to the magnetopause. In the last case, the spacecraft crosses the magnetopause while observing the FTE. (Adapted From Elphic (1995))

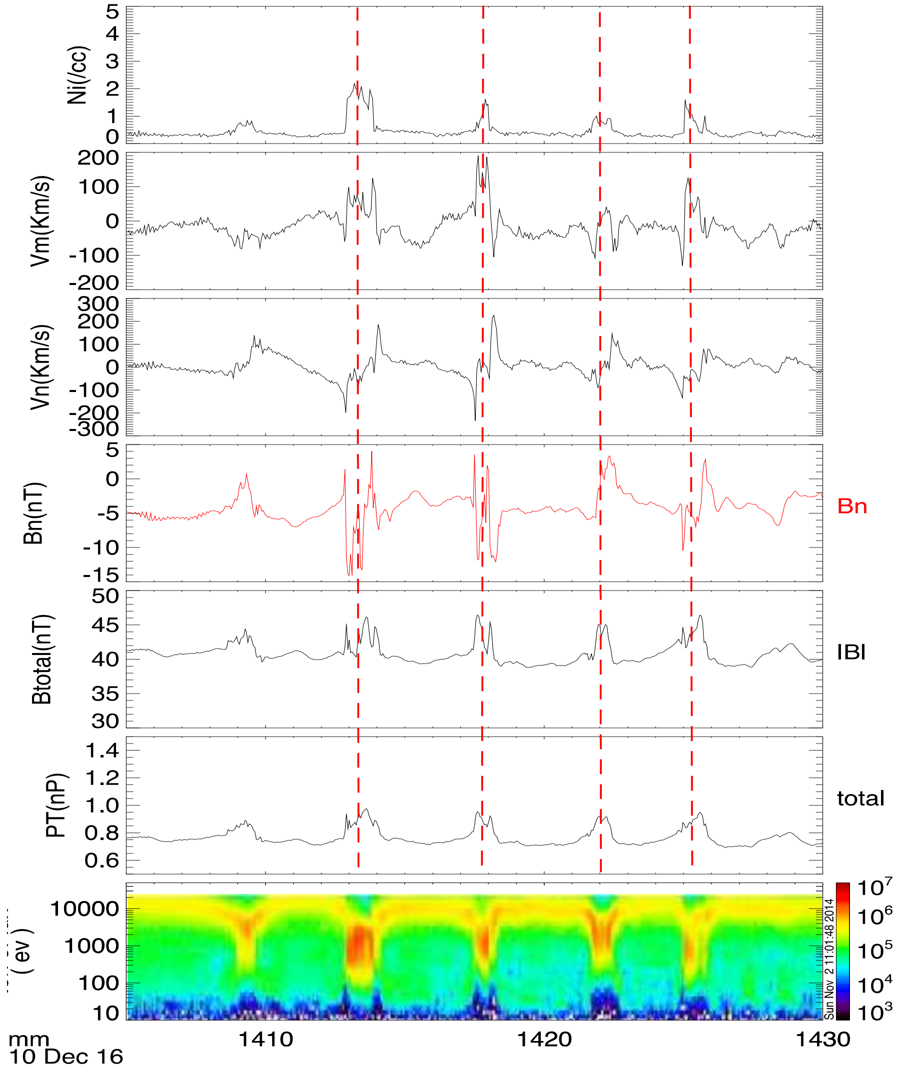

Supplementary Figure 3. Themis D Observation of FTEs on 16 Dec 2010. From top to bottom: (a) Ion density, (b) M component of the velocity V_M, (c) N components of the velocity V_N, (d) N component of magnetic field B_N (e) Magnetic field magnitude |B|, (f) total (magnetic plus ion) pressure, (g) omnidirectional ion energy flux spectrogram. The red vertical dashed lines mark the approximate centers of the bipolar structures. Themis D observed quasi-periodic fluctuations at the dawn flank MP during the interval 14:09 – 14:25 UT. The solar wind had a flow speed of 500 km/s and a density of 9 cm^{-3}, while the IMF vector was (-1,-2,-2) nT. There were no significant solar wind dynamic pressure variations before or during the event. THEMIS D was located at (7.5,-7.8,4.3), i.e., near 9 MLT and was moving anti-sunward. The structures show bipolar B_N pulses and all other expected FTE signatures.

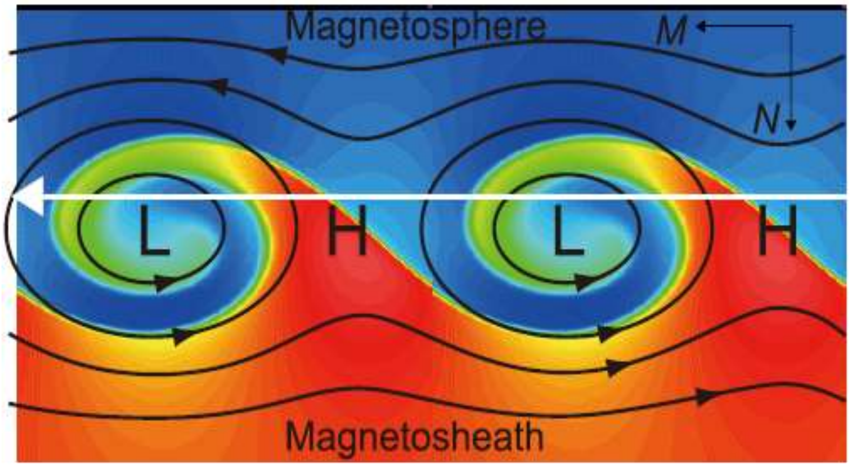

Supplementary Figure 4. Schematic drawing of rolled-up KH vortices at the dusk-flank magnetopause. Schematic of the relationship between the streamline pattern (black lines) and total (magnetic plus plasma) pressure and density (red, dense; blue, tenuous) distributions, when viewed in the vortex rest frame (from Hasegawa et al., 2012). The sub-solar region is to the left. The total pressure minimizes at the center (L) of the vortices, while it maximizes at the hyperbolic point (H), which is a flow stagnation point in the vortex rest frame, and around which the streamlines form hyperbolas. It is expected that magnetosphere-to-magnetosheath transitions can be characterized by large and rapid density increases that approximately coincide with maxima in the total pressure.

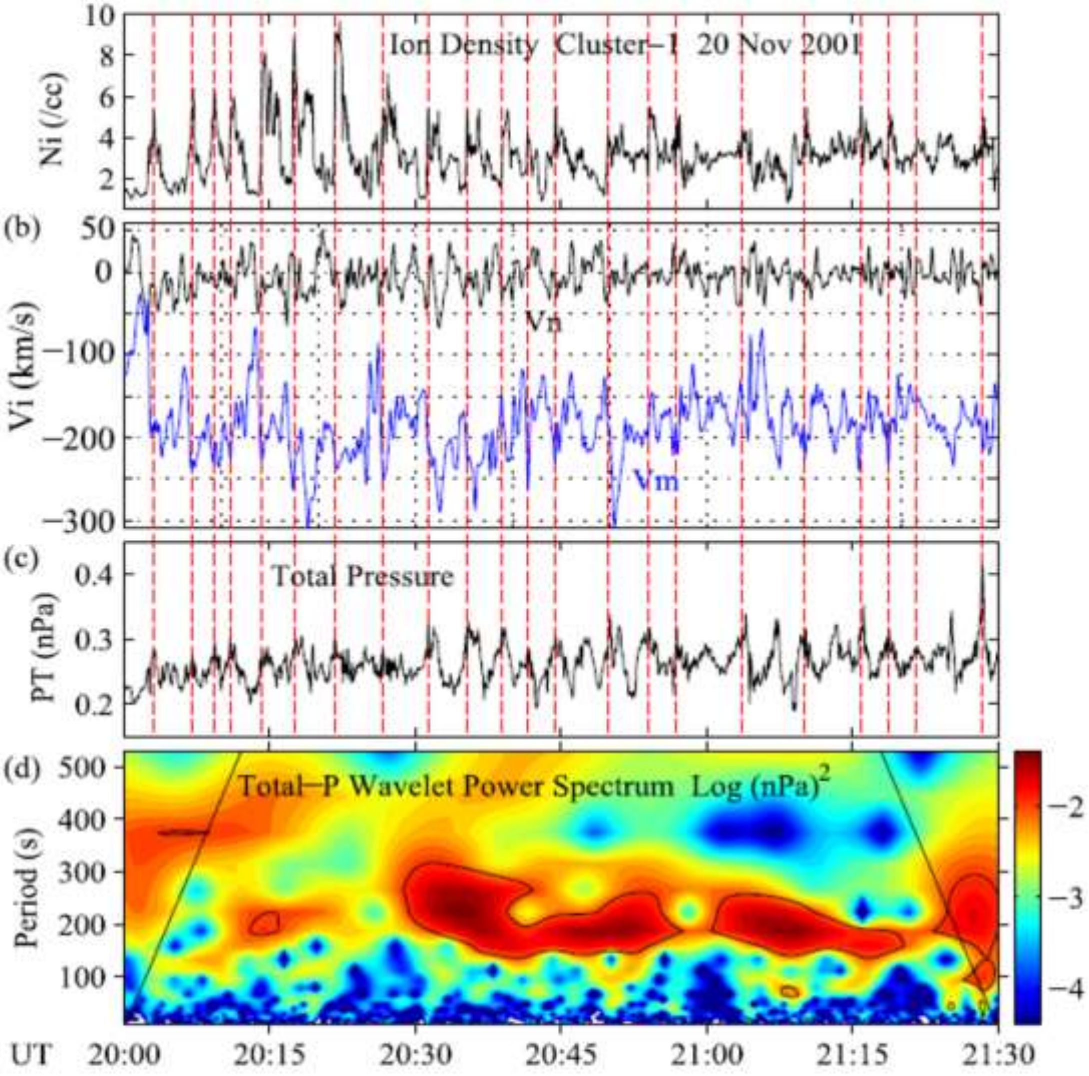

Supplementary Figure 5. Breaking KHW observed by Cluster. Quasi-periodic fluctuations of the bulk plasma parameters during Cluster 1 (C1) observations of rolled-up KH vortices showing: (a) ion density, (b) M and N components of the smoothed velocity (c) total pressure (magnetic plus ion pressure), and (d) wavelet spectra of the total pressure (from Hasegawa et al., 2012.) Note that density jumps from the magnetospheric to magnetosheath values closely coincide with total pressure maxima (red vertical dashed lines), as expected for KH vortices in the non-linear stage.

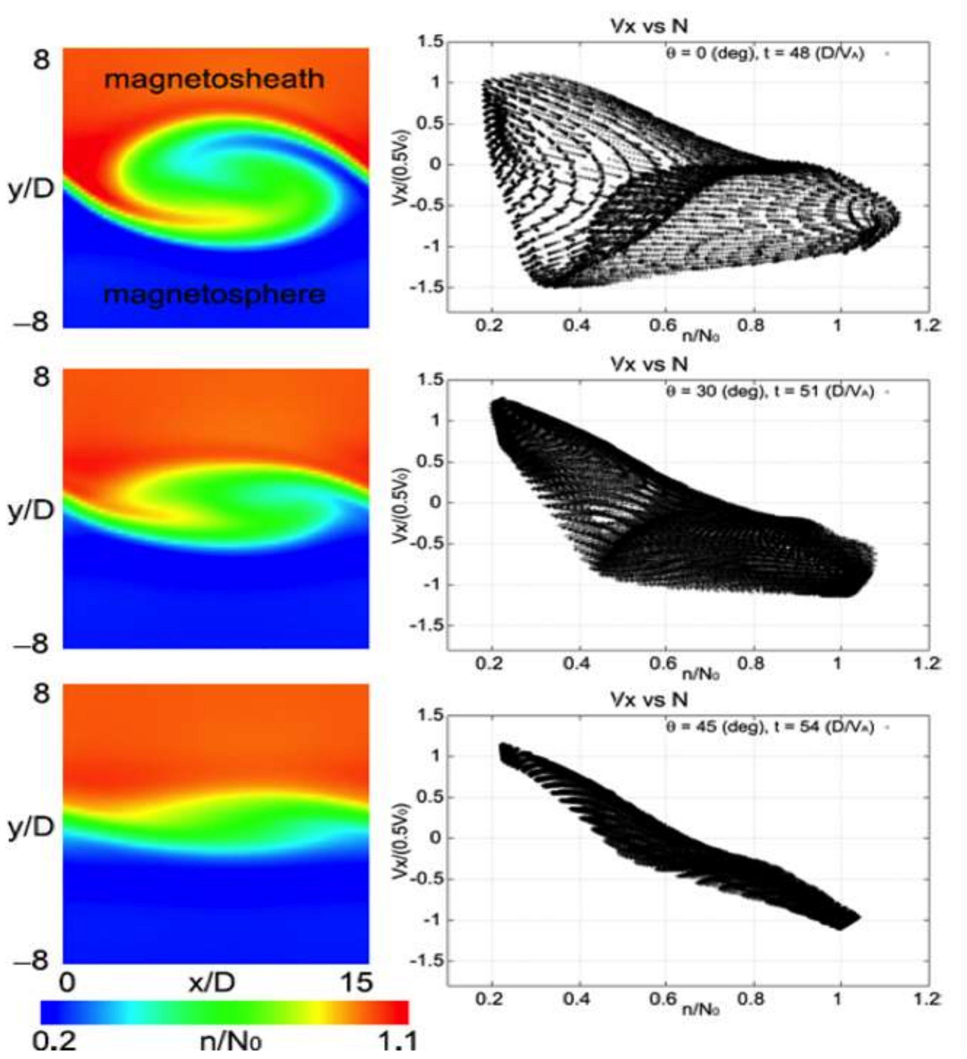

Supplementary Figure 6. MHD simulation of KH waves. Plasma density (left column) and scatter plots of the x (sunward) component of the velocity V_X versus plasma density N/N_0, at three different stages from 3D MHD KHW simulations (from Hasegawa et al., 2006.) The left panels show the normalized density in the x-y plane in color. The initial values of V_X are −1 and +1 on the magnetosheath and on the magnetospheric side, respectively. The scatter plots were generated from virtual spacecraft observations of the simulated KH wave or vortex, and exhibit a distinct pattern, depending on the phase of the KHW growth. Within the rolled-up vortex (top), a significant fraction of low-density plasma ($N/N_0 < 0.5$) has an anti-sunward speed higher than that of the magnetosheath plasma characterized by $V_X = -1$ and $N/N_0 = 1$. In the linear and not-yet-rolled-up stages (bottom two rows) no such relation exists.

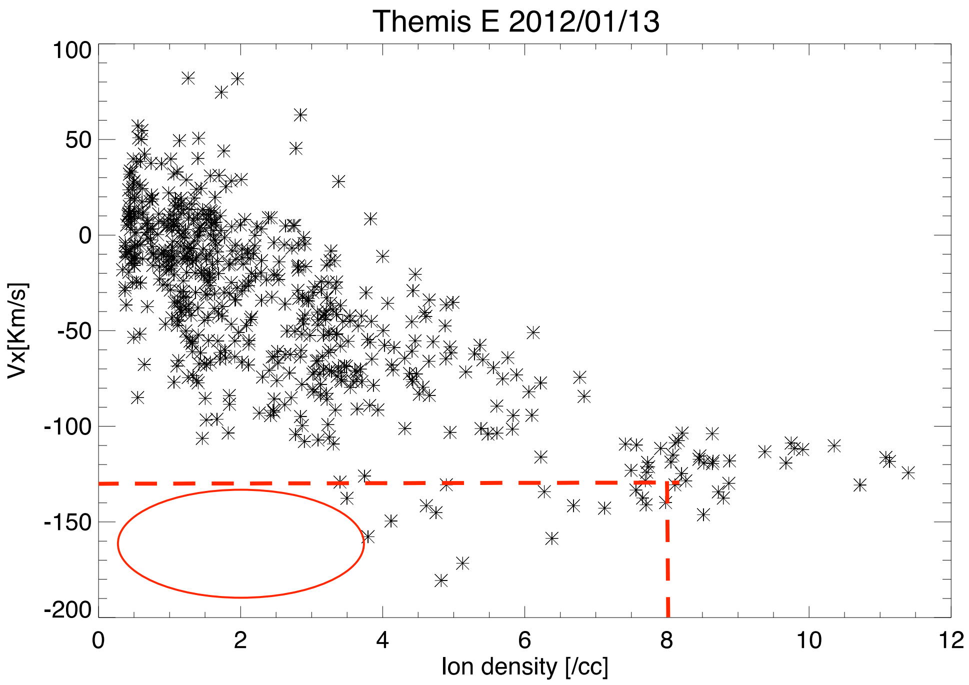

Supplementary Figure 7. Scatter plot of the velocity component, V_X versus ion density for KHW in the linear stage on 13 Jan 2012 detected by THEMIS E. The −x direction is anti-sunward and is roughly along the −M direction, tangential to the magnetopause. The dashed lines show the magnetosheath velocity and the average magnetosheath plasma density. The plot confirms that there is no low-density plasma with anti-sunward speed higher than that of the magnetosheath plasma, which would be expected within the red oval. Therefore, we conclude that this event shows KHW that are between the linear and non-linear stages, i.e., the waves are not yet rolled up.

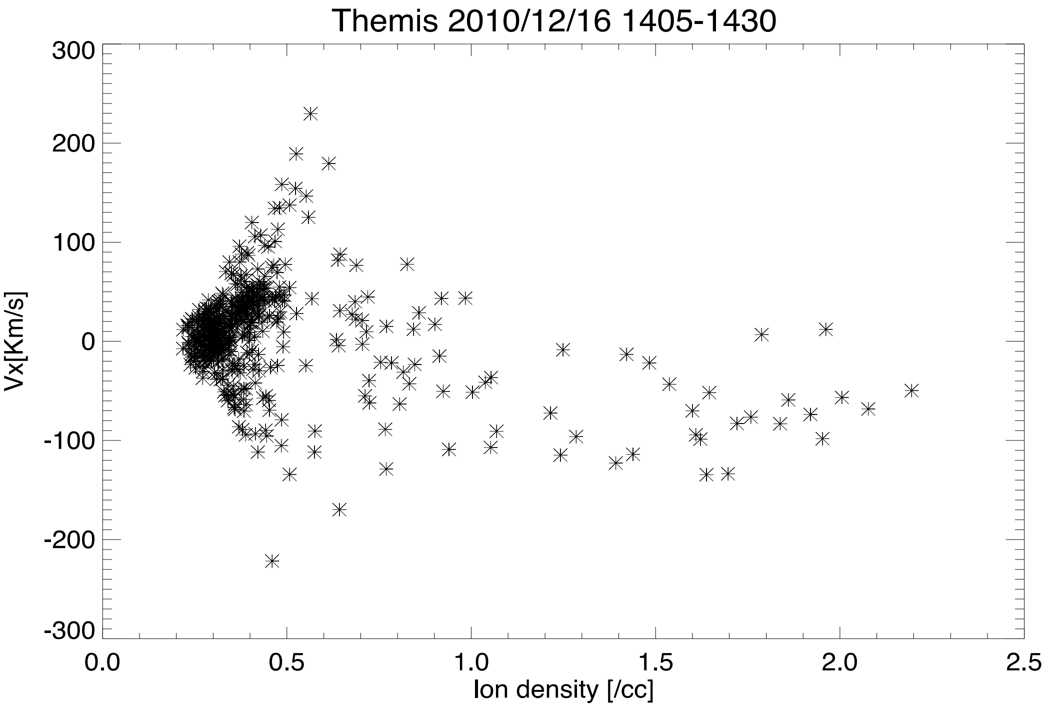

Supplementary Figure 8. Scatter plot of velocity, V_X versus ion density, N, for FTEs detected by THEMIS D on 16 December 2012. This scatter plot shows that FTEs always fail the V_X – N test. There are also significant tangential flows associated with the FTEs, but these occur mostly at the edges of the FTEs. These flows show that FTEs can basically be viewed as solid structures that plow through the background plasma along the magnetopause. However, despite these strong flows, the tangential flow speed never exceeds the magnetosheath flow speed. The scatter plot pattern for this event thus looks very different than those produced by KHW. Specifically, the FTE pattern shows more sunward flows, which arise from the flow of plasma around the FTE to fill the void behind it.

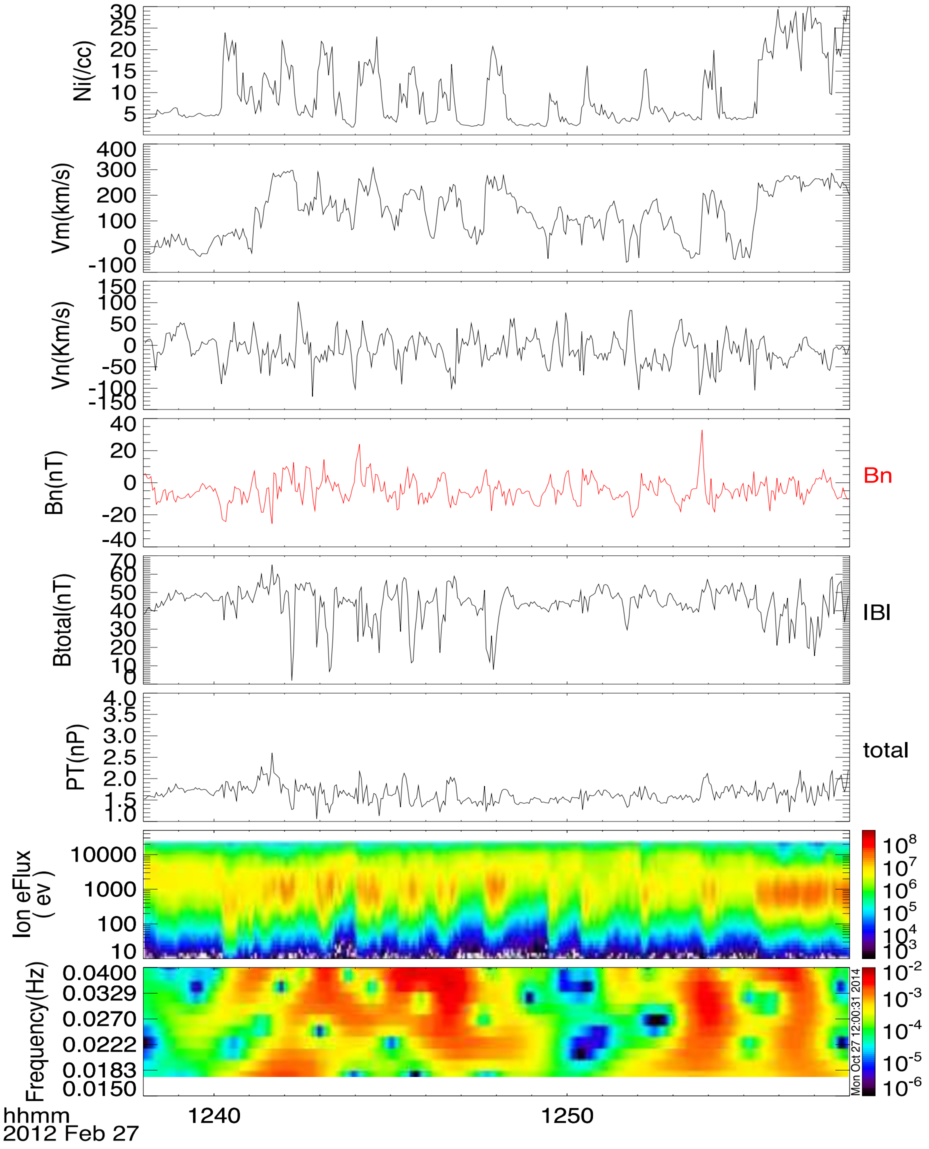

Supplementary Figure 9. THEMIS A observations of KHWs under southward IMF on 27 Feb 2012. From top to bottom: (a) ion density, (b) M component of the velocity V_M, (c) N components of the velocity V_N, (d) N component of magnetic field B_N, (e) magnetic field magnitude |B|, (f) total (magnetic plus ion) pressure, (g) omnidirectional energy flux, and (h) wavelet spectra of the total pressure. The solar wind had a flow speed of 500 km/s, a density of N = 15 cm^{-3}, and the IMF vector was (-2,-3,-5) nT, i.e., the IMF was southward. There were no significant solar wind dynamic pressure variations before or during the event. THEMIS A was located at (-0.2,-10.6,2.8), i.e., near the dawn terminator, and was moving sunward. The event is characterized by continuous fluctuations in the magnetic field components, the velocity components, and the ion density. Although some of the B_N fluctuations look bipolar, like in FTEs, they are not associated with significant maxima in the total pressure. Also, the total magnetic field does not have the maxima or crater-like signatures centered on the B_N=0 traversals, which are typical for FTEs. On the contrary, the field magnitude has very distinct deep minima, which are not seen in conjunction with FTEs.

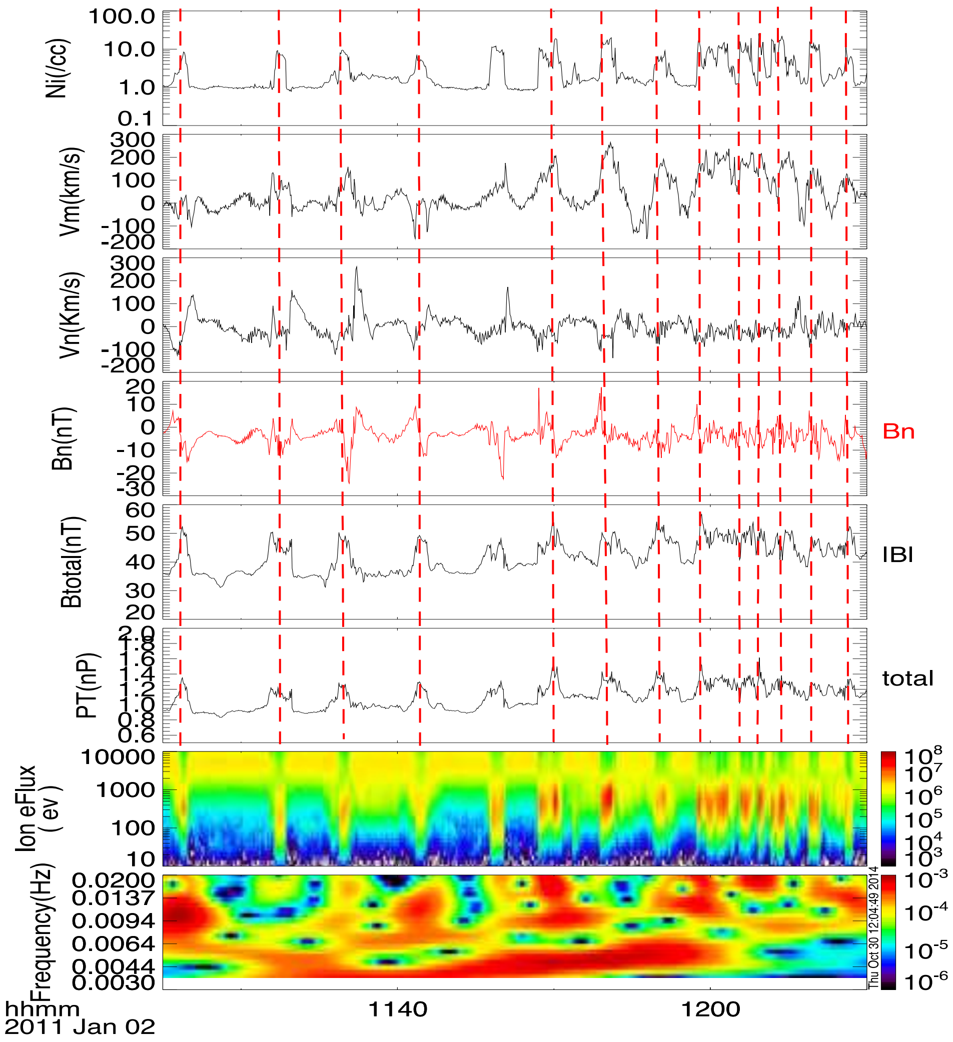

Supplementary Figure 10. **THEMIS E observations of FTEs and KHWs on 2 January 2011.** From top to bottom: (a) ion density, (b) M component of the velocity V_M, (c) N components of the velocity V_N, (d) N component of magnetic field B_N, (e) magnetic field magnitude |B|, (f) total (magnetic plus ion) pressure, (g) ion energy flux, and (h) wavelet spectrum of the total pressure. The solar wind had a flow speed 330 km/s; density of 12 cm^{-3}, and the IMF vector of (4,4,2) nT. THEMIS E crossed the magnetopause about 3 R_E sunward of the dawn terminator. The characteristics of the fluctuations changed from the 11:30 - 11:55 UT interval to the 11:55 - 12:10 UT interval. Specifically, during the first sub interval the fluctuations are separated by several minutes of quiet each, while the second part of the event is characterized by continuous fluctuations, which also have shorter periods, i.e., ~250 second versus ~100 seconds. The first part of the event clearly consists of FTEs, It also has the other FTE signatures such as the maxima of total pressure and magnetic field strength at the centers of the bipolar B_N excursions, indicated in the plot by vertical red dashed lines. Some of the FTEs can be characterized as crater-FTEs, exhibiting a W-shaped structure of the field magnitude. For the second part of the event, the density jumps indicate traversals from the magnetosphere to the magnetosheath (marked dashed red lines) that closely coincide with total pressure maxima, which are also less pronounced than the total pressure maxima of the first sub interval. These maxima are located right at the edges of the density structures, which is expected for rolled-up KH vortices. The existence of B_N excursions that look somewhat bipolar also supports the interpretation that these are rolled-up KH vortices. In contrast to the FTEs of the first sub interval, these B_N signatures are smaller and more irregular.

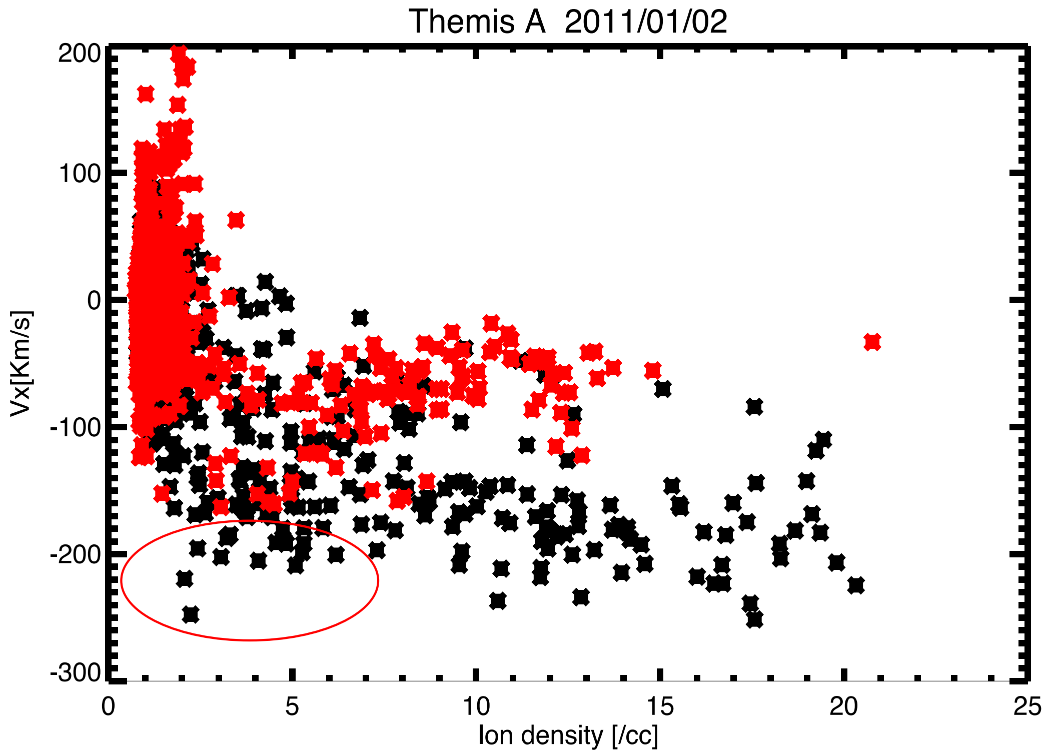

Supplementary Figure 11. Scatter plot of the velocity V_X versus ion density for FTE/KHW event on 2 January 2011 detected THEMIS E. The −x direction is defined as in Supplementary Figure 8. The red symbols show the first part of the event (11:20-11:50 UT), which were identified as FTEs. The black symbols show the second part of the event (11:55-12:10 UT), which were identified as KHW. There is some low-density plasma (circled in red) that flows faster than the magnetosheath plasma (~-200 km/s), confirming that rolled-up vortices are present. However, during the FTE interval the tangential flows are mostly less than 100 km/s, which clearly separates the FTEs from KHW.

Supplementary Table 1 . Summary of characteristic FTE times

Reference	FTE characteristic times
Rijnbeek et al. (1984) [4]	"The typical duration of FTEs was found to be ~1-2 min. … FTEs observed in the magnetosheath and magnetosphere have similar recurrence times of ~7 and ~8 min, respectively."
Elphic (1990) [5]	"These disturbances (FTEs) are brief (1-2 minutes) and separated by a longer period of quiet (typically 6 - 9 minutes)."
Lockwood & Wild (1993) [6]	"Magnetopause observation by the ISEE satellites shows that the distribution of the intervals between FTE signatures has a mode value of 3 min. The mean value is found to be 8 min."
Elphic (1995) [1]	"In the case of FTEs possessing the requisite reconnection behavior the evidence points toward single x-line reconnection modulated at some characteristic (1-2 minute) time scale, with a recurrence interval of several times the modulation time scale (5-10 minutes)."
Kuo et al. (1995) [7]	Obtained 10.5 min (median: 8 min) FTE separation time from their ISEE 1 FTE study.
Neudegg et al. (2000) [8]	Using Equator-S data, found FTEs with an average FTE separation time of 8.8 min, assuming intervals larger than 20 min are not part of the same reconnection sequence.
Wang & Elphic (2005) [9]	"We obtain an average FTE separation time of 7.09 min, which is at the lower end of the previous results."

Supplementary Table 2. Comparison between the properties of FTEs and Kelvin-Helmholtz Vortices

Signature	Kelvin-Helmholtz Vortex	FTE
Magnetic field	1. Bipolar B_N. 2. Often has a maximum in magnetic field strength at the edge of the vortex, with less than 10 nT magnitude. 3. Continuous bipolar B_N.	1. Bipolar B_N. 2. Has a magnetic field strength maximum at the core of FTE, usually larger than 10 nT magnitude. 3. Bipolar B_N separated by a few minutes quiet.
Plasma	1. Substantial pressure perturbations, minimum at the vortex center and maximum at the edge. A large and rapid density increase coincides approximately with a maximum in the total pressure at the edge of the vortex. 2. Usually small perturbation in V_N. 3. Low-density plasma flowing faster than sheath velocity.	1. Total pressure maxima at the FTE center. 2. Typically bipolar V_N .The V_N perturbation is usually larger than those seen in KHW. 3. No accelerated low-density plasma.
Duration and Period	1. Continuous wave trains. 2. 1-4 minute periods.	1. Short (1-2 min) bipolar B_N signatures separated by quiet. 2. Repetition period typically longer than 4 minutes.

Supplementary References

1. Elphic, R. C., Observations of Flux Transfer Events: A Review. *Physics of the Magnetopause*, Geophys. Monogr. Ser., **90**, 225 - 233 (1995).

2. Hasegawa, H., Structure and dynamics of the magnetopause and its boundary layers, *Monogr. Environ. Earth Planets*, **1**, 71 - 119 (2012).

3. Hasegawa, H., et al., Single-spacecraft detection of rolled-up Kelvin-Helmholtz vortices at the flank magnetopause, *J. Geophys. Res.*, ***111***, A09203 (2006).

4. Rijnbeek, R. P., et al., A survey of dayside flux transfer events, observed by the ISEE-1 and -2 magnetometers, *J. Geophys. Res*., **89**, 786 - 800(1984).

5. Elphic, R. C., Observations of flux transfer events: Are FTEs flux ropes, islands, or surface waves? *Physics of Magnetic Flux Ropes*, Geophys. Monogr. Ser., **58**, 455 - 471 (1990).

6. Lockwood, M., and M. N. Wild, On the quasi-periodic nature of magnetopause flux transfer events, *J. Geophys. Res.,* **98**, 5935–5940 (1993).

7. Kuo, H., C. T. Russell, and G. Le, Statistical studies of flux transfer events, *J. Geophys. Res*., **100**, 3513 - 3519 (1995).

8. Neudegg, D. A., et al., A survey of magnetopause FTEs and associated flow bursts in the polar ionosphere, *Ann. Geophys*., **18**, 416 - 435 (2000).

9. Wang, Y., et al., Initial results of high-latitude magnetopause and low-latitude flank flux transfer events from 3 years of Cluster observations, *J. Geophys. Res*., **110**, A11221 (2005).
